# Maternal perinatal mental health and offspring academic achievement at age 16: the mediating role of childhood executive function

**DOI:** 10.1111/jcpp.12483

**Published:** 2015-11-29

**Authors:** Rebecca M. Pearson, Marc H. Bornstein, Miguel Cordero, Gaia Scerif, Liam Mahedy, Jonathan Evans, Abu Abioye, Alan Stein

**Affiliations:** ^1^ School of Social & Community MedicineUniversity of BristolBristolUK; ^2^ Section of Child & Adolescent PsychiatryDepartment of PsychiatryUniversity of OxfordOxfordUK; ^3^ Child and Family ResearchEunice Kennedy Shriver National Institute of Child Health and Human DevelopmentBethesdaMDUSA; ^4^ Department of Experimental PsychologyUniversity of OxfordOxfordUK; ^5^ Institute of Psychological Medicine and Clinical NeurosciencesCardiff UniversityCardiffUK; ^6^ School of Public HealthUniversity of WitwatersrandJohannesburgSouth Africa

**Keywords:** ALSPAC, postnatal depression, prenatal anxiety, executive function, academic achievement, math

## Abstract

**Background:**

Elucidating risk pathways for under‐achieving at school can inform strategies to reduce the number of adolescents leaving school without passing grades in core subjects. Maternal depression can compromise the quality of parental care and is associated with multiple negative child outcomes. However, only a few small studies have investigated the association between perinatal maternal depression and poor academic achievement in adolescence. The pathways to explain the risks are also unclear.

**Method:**

Prospective observational data from 5,801 parents and adolescents taking part in a large UK population cohort (Avon‐Longitudinal‐Study‐of‐Parents‐and‐Children) were used to test associations between maternal and paternal depression and anxiety in the perinatal period, executive function (EF) at age 8, and academic achievement at the end of compulsory school at age 16.

**Results:**

Adolescents of postnatally depressed mothers were 1.5 times (1.19, 1.94, *p* = .001) as likely as adolescents of nondepressed mothers to fail to achieve a ‘pass’ grade in math; antenatal anxiety was also an independent predictor of poor math. Disruption in different components of EF explained small but significant proportions of these associations: attentional control explained 16% (4%, 27%, *p* < .001) of the association with postnatal depression, and working memory explained 17% (13%, 30%, *p* = .003) of the association with antenatal anxiety. A similar pattern was seen for language grades, but associations were confounded by maternal education. There was no evidence that paternal factors were independently associated with impaired child EF or adolescent exams.

**Conclusion:**

Maternal postnatal depression and antenatal anxiety are risk factors for adolescents underachieving in math. Preventing, identifying, and treating maternal mental health in the perinatal period could, therefore, potentially increase adolescents’ academic achievement. Different aspects of EF partially mediated these associations. Further work is needed, but if these pathways are causal, improving EF could reduce underachievement in math.

## Introduction

Math and language skills are a strong determinant of employment, health, and social functioning worldwide. In the United Kingdom, for example, without achieving a ‘pass’ (A*–C grade) in math and English exams at the end of compulsory education, adolescents will not be considered for higher education and their employment prospects are poor (Wolf, 2011). In 2013, over one‐quarter of adolescents in the United Kingdom left school without these grades (Wolf, 2011). This circumstance represents a substantial economic burden (Wolf, 2011), which is echoed internationally (OECD, 2012). Identifying and understanding early risk factors for underachieving in math and language are, therefore, important foci for research. School‐level contextual factors explain surprisingly little variance in academic achievement (Nye, Konstantopoulos, & Hedges, 2004); even in high‐quality and universally available education systems, a significant proportion of adolescents fail to reach pass grades. It is therefore important to understand the role of individual level risk factors.

Maternal postnatal depression (PND) is common (10–15%; Gavin et al., 2005), can disrupt parenting processes, and increases the risk of a range of negative child outcomes, including impaired cognitive development in childhood (Hughes, Roman, Hart, & Ensor, 2013; Murray, Halligan, & Cooper, 2010; Murray et al., 2011). However, only one study has investigated whether PND is associated with end of high‐school achievement (Murray et al., 2011). PND is often a continuation of antenatal depression symptoms and is linked with closely related symptoms of anxiety during pregnancy. Antenatal symptoms have also been linked to long‐term cognitive outcomes in the child, such as IQ (Evans et al., 2012), possibly due to programming effects on fetal neuro‐development (Glover, O'Connor, & O'Donnell, 2010). However, no study has investigated the impact of prenatal symptoms on end of high‐school achievement.

Therefore, further investigation of this association from large prospective studies is needed. If an association between PND or antenatal anxiety and academic achievement were established, identifying modifiable, intermediate factors through which such exposures exert effects would present important opportunities for early intervention.

### Executive function as a mediating pathway

We hypothesize that disruptions to the child's development of executive functions (EFs) represents one modifiable pathway to academic achievement from both perinatal depression and anxiety risk factors. The EFs are general‐purpose control processes that regulate thoughts and behaviors (Miyake & Friedman, 2012; Miyake et al., 2000). Early life adversity, including disrupted or absent maternal care, is associated with impaired development of EF in childhood (Hackman, Farah, & Meaney, 2010; Hostinar, Stellern, Schaefer, Carlson, & Gunnar, 2012). Evidence in animals has directly shown that poor maternal care and social deprivation lead to poor performance on EF tasks and alterations in brain regions involved in EF, such as the medial prefrontal cortex (Hostinar et al., 2012; Monroy, Hernandez‐Torres, & Flores, 2010). Despite widely documented evidence that maternal depression is associated with disruptions to maternal care (Field, 2010), to our knowledge only a few studies have linked maternal depression to impaired EF (Comas, Valentino, & Borkowski, 2014; Hughes et al., 2013) and only one linked prenatal anxiety with EF (Buss, Davis, Hobel, & Sandman, 2011). Impaired EF in childhood is in turn predictive of poor academic achievement (Bull, Espy, & Wiebe, 2008; Clark, Sheffield, Wiebe, & Espy, 2013). These associations provide evidence that disruption to EFs could be one mediating pathway from maternal depression and anxiety to academic achievement, but this chain has not been directly tested. We do so here. EFs provide important components of the pathway to target because of evidence that they are amenable to modification in childhood (Diamond & Lee, 2011).

### Aims of study

We used data from over 5,000 mothers and adolescents taking part in a large UK population cohort to test the hypothesis that maternal PND and anxiety are associated with increased risk of children's underachieving in math and language exams at the end of high‐school. We also estimated the extent to which any associations are mediated by indirect pathways through different components of EF in childhood. We focused on the three related but separable components of EF: cognitive flexibility or attentional switching (switching between information), attentional control or inhibition (maintaining focus and inhibiting prepotent responses), and updating or working memory (holding and updating information for current processing) (Miyake & Friedman, 2012, Manly et al., 2001). Examining the relative importance of EF components is necessary to ensure that interventions target the most appropriate component(s). To separate these three EFs of interest from related cognitive capacities that influence performance on the EF tasks, we investigated the roles of selective attention (attending to target stimuli among distractors), processing speed (speed at which the child can read out words or put pen to paper), and IQ.

Finally, to assess the specificity of any maternal effects, we investigated the role of paternal factors. Paternal comparisons offer important ‘negative controls’ because fathers provide equivalent genetic contributions to offspring and usually share the family environment (income, neighborhood); however, in most cases fathers spend less time with their infants than mothers, have a reduced role in parenting (Bornstein, 2015), and do not carry the fetus. Thus, if the impact of PND or anxiety on parenting or fetal programming explains associations, we would observe no effects of paternal depression/anxiety. In contrast, if genetic or environmental confounding explains associations with maternal factors, equivalent associations would be expected for equivalent paternal factors.

## Methods

### Sample

The sample comprised participants from the Avon‐Longitudinal‐Study‐of‐Parents‐and‐Children (ALSPAC) which recruited 14,541 pregnant mothers resident in United Kingdom with expected delivery between 1/4/1991 and 31/12/1992 (Fraser et al., 2013), over 70% of the eligible population. The study has continued to follow parents and offspring. Ethical approval for the study was obtained from the ALSPAC Law and Ethics Committee and Local Research Ethics Committees, and participants gave informed consent. More detailed information on the ALSPAC study is available on the study website which contains details of all the data that are available through a fully searchable data‐dictionary (http://www.bris.ac.uk/alspac/researchers/data-access/data-dictionary/). The current study uses data from the sample (singletons only) attending a face‐to‐face assessment clinic at age 8 and with consented linked exam data at age 16.

**Figure 1:**
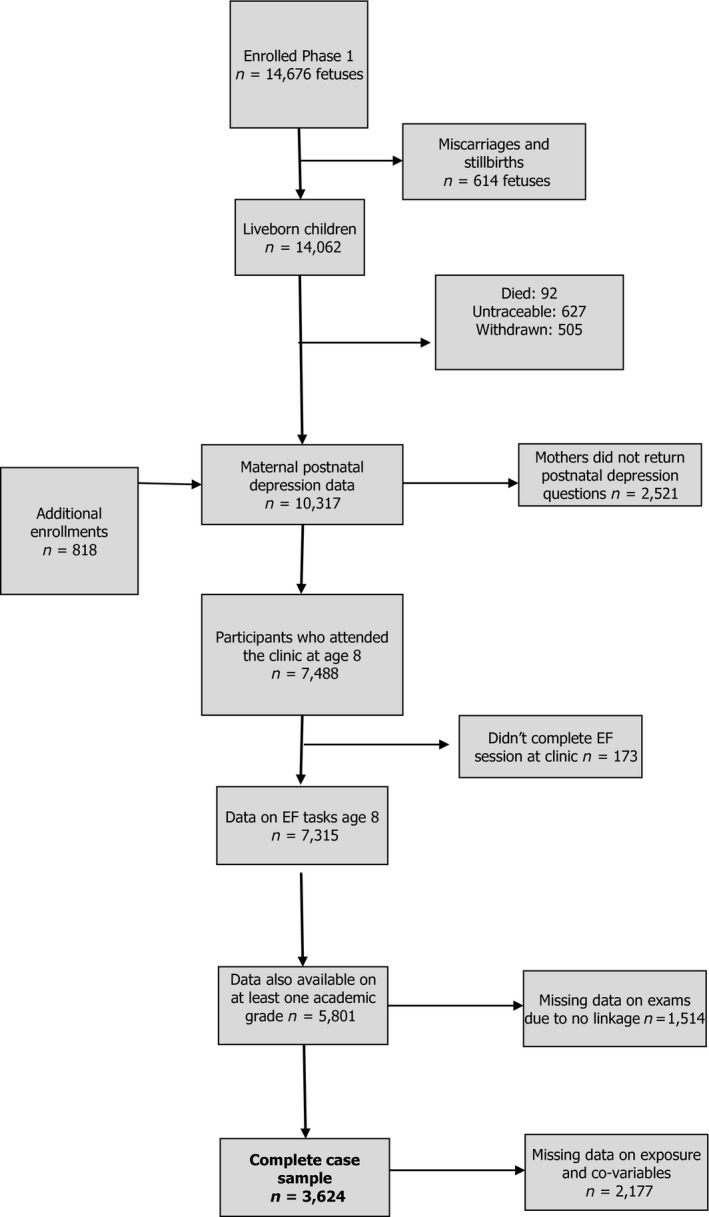
Flow chart indicating sample size and sources of missing data

Our starting sample was mothers who completed maternal depression postal questionnaires for PND (*n *=* *10,317). Of this sample, 7,557 children completed EF tasks at age 8, and exam data were available for 6,404. A sample with complete data across all exposure, outcome, mediating, and confounding variables (*n *=* *3,624) was primarily used. However, where missing values were predictable by observed data from other time points, missing data were imputed (see later), and all analyses were repeated using the same sample (*n *=* *5,801; Figure 1).

### Measures

#### Exposure

Symptoms of maternal and paternal depression were measured using the Edinburgh‐Postnatal‐Depression‐Scale (EPDS; Cox & Holden, 2003). The EPDS is a 10‐item self‐report depression questionnaire validated for use in and outside of the perinatal period (Cox & Holden, 2003). Postal questionnaires, including EPDS measures, were administered at approximately 8 weeks and 8 months postnatally and again when the child was 1.5 and 2.5 years of age. To make full use of the variation in symptoms and maximize power, we primarily use continuous scores for statistical analyses, averaging EPDS scores across the two available measures within the postnatal period to give a more stable and reliable estimate of symptoms over that period (Pearson et al., 2013). However, in descriptive analyses to aid interpretation and clinical relevance, we created a categorical variable (Appendix S1). Maternal anxiety was measured in pregnancy using the anxiety items from the Crown–Crisp index, a validated self‐rating inventory (Birtchnell et al., 1988; Sutherland & Cooper, 1992).

#### Outcome (age 16)

With consent from participants, grades achieved in Mathematics and English Language were extracted from external national educational records. Binary variables were created to represent reaching an A*–C grade in math and English language (coded as 0) or not (coded as 1). Continuous scores were also derived (1–10, with grade A* representing 1 and grade U, unclassifiable – the lowest grade, representing 10).

#### Mediating‐variables (age 8)

A number of established cognitive tasks were used to measure three core EFs. All measures were derived in accordance with the original manual, and full details of the tasks are provided in Appendix S1.

#### Updating/working memory

(a) The digit‐span memory task from the Wechsler‐Intelligence‐Scale‐for‐Children (WISC‐IIIUK): children were presented with a string of numbers in forwards and backwards order and asked to repeat them in the order of presentation. The digit‐span score was the sum of items correctly recalled, the WISC‐III UK has good test‐retest reliability (.80–.89) (Strauss, Sherman, & Spreen, 2006). (b) An adaptation of the Nonword Repetition Test. This task comprised 12 nonsense words. The child listened to each word via an audio cassette recorder and then repeated each.

#### Attentional switching

The dual‐attention task of ‘Sky Search’ subtest of the adapted Test‐of‐Everyday‐Attention‐for‐Children (TEA‐Ch; Robertson, Ward, Ridgeway, & Nimmo‐Smith, 1996) was used. The child initially selected pairs of spaceships from a task sheet containing matching and nonmatching spaceships. The task was repeated but with the addition of another task: the child was also requested to count the number of noises played during the task. The difference in speed and accuracy when completing the task with and without the addition of noises was taken as an indication of switching. This measure has good test–retest reliability (*r *=* *.81; Manly et al., 2001).

#### Attentional control

The inhibition aspect of the ‘Opposite Worlds’ task from the TEA‐Ch was used. This is a basic form of a ‘Stroop’ task, where the child is required to give a verbal response that contradicts the visual information given. The child was presented with a trail of digits and instructed to read out ‘one’ when presented with a 2 and ‘two’ when presented with a 1. Time to complete the task was taken as the measure of attentional control. This measure has good test‐retest reliability (*r *=* *.92; Manly et al., 2001).

#### Processing speed: verbal and motor

Time taken to complete the baseline condition of the ‘Opposite Worlds’ Task (time taken to read the trail of numbers as ones and two, i.e., the ‘same worlds condition’) was taken as the measure of verbal processing. Motor‐processing speed was taken as the time and accuracy to circle the spaceships in a different ‘Sky Search’ task sheet with only identical pairs.

#### Selective attention

The baseline condition of the ‘Sky Search’ task was used, how fast and accurately the child selected pairs of spaceships from the task sheet containing matching and nonmatching spaceships (without the addition of the noises). This measure has good test‐retest reliability (*r *=* *.90; Manly et al., 2001).

#### IQ was measured using the WISC‐IIIUK at age 8

See Appendices S1 and S2.

### Confounding variables

Variables associated with both PND and child outcomes, not considered part of the causal pathway, were included in regression models. *Maternal‐characteristics*: Age at birth of the index child, parity, social class, education, IQ, and maternal depression close to the time of the cognitive assessments (age 7, using the EPDS). *Child‐characteristics*: Birth weight, gestational age, and child‐reported enjoyment of school at age 8 (ordinal‐rating scale).

### Analysis

#### Main effects

A series of separate linear regression models were conducted to investigate associations between continuous PND scores in mothers and fathers with each of the three measures of EFs, the three general cognitive abilities, and exam grades (using logistic regressions for binary ‘pass/no‐pass’ outcomes). Analyses were conducted before and after adjustments for confounding variables.

#### Mediation (indirect effects)

We investigated mediation through path analysis, quantifying: (a) direct pathways between PND and exam grades and (b) indirect pathways through any cognitive measures which were associated with PND. Mplus applies the product‐of‐coefficients strategy in the assessment of indirect effects (MacKinnon, Fairchild, & Fritz, 2007). We modeled the associations between all variables in a combined model, where the correlations between each EF measure and the correlation between math and English grades were taken into account (Appendix S3).

#### Investigation of prenatal effects

In a further analysis we adjusted for antenatal anxiety. We adjusted for antenatal anxiety rather than antenatal depression because our aim was to account for potentially different pathways of biological programming effects in utero, rather than capturing the same variance as PND (which, given the strong association between the EPDS in pregnancy and postnatally, *r *>* *.7, could be the case when adjusting for symptoms of antenatal depression). Anxiety is most closely related with an increased stress response, such as increased cortisol, whereas a more complex relation is found in depression with evidence for reduced stress responding (in some cases). Therefore, antenatal anxiety more closely accounts for the in utero stress mechanism. Antenatal anxiety is still highly correlated with PND (*r *=* *.5), but lower than the correlation between antenatal and PND, allowing for some separation of independent effects. Although there was little variation in population‐level symptoms of depression from the antenatal and postnatal periods, clinically and theoretically meaningful differences may reside in the effects of ‘episodes’ of particularly high symptoms of depression. To account for any effects of particularly high symptoms of depression in pregnancy, we excluded those mothers with clinically relevant symptoms antenatally (>12 on EPDS).

#### Missing data

We used the substantial information on sociodemographic variables, which predict both the missing variables and the pattern of missing data, to impute. The chosen method assumes data are Missing at Random, whereby any systematic differences between the missing and the observed values can be explained by differences in observed data. We feel confident in this assumption because the differences between participants with complete and incomplete data have been investigated in detail elsewhere (Fraser et al., 2013). There is evidence that participants with missing data are from lower socio economic status and measures such as income, education, and house ownership are available to use in imputation models (Fraser et al., 2013; Appendix S4).

## Results

The mean maternal EPDS depression score postnatally was 5.5 (*SD* = 4.4, range = 0–27). Ten percent of mothers exceeded the EPDS threshold for depression at the 8 weeks wave. Of these mothers 6% exceeded the EPDS threshold on at least one more occasion in the next 2 years, see Pearson et al. (2013) for further description of this sample.

### Main effects

#### PND and EF

Of the three EFs there was evidence from linear regression models that PND was associated with impairments in attentional control and switching (Tables 1 and 2). There was also evidence for a dose–response effect, where increasing levels of PND were associated with greater impairment, particularly for attentional control. Adolescents of mothers with persisting PND were .23 *SD* slower (.10 to .40, *p* = .001) than adolescents of non‐depressed mothers in completing the attentional‐control task (see Table S1). These associations remained following adjustments for confounding variables, after removing mothers with antenatal depression, and following imputation for missing data. However, there was no consistent evidence for an association between PND and working memory. Of the related cognitive abilities, there was no evidence that PND was associated with motor‐processing or selective attention; however, PND was associated with verbal processing. Verbal processing was thus controlled for in the mediation model.

**Table 1:** Linear regression models investigating the association between postnatal depression scores and executive function and general cognitive abilities at age 8, before and after adjustments

	Association of EPDS continuous score (postnatal average) to each executive function outcome, standardized regression coefficient (95% CI) (*n *= 3,624)			Adjusted for maternal IQ in the subsample with available data about both maternal IQ^b^ and child measures (*n *= 1,471)	Postimputation and including adjustments (*n *= 5,801)
	Unadjusted (*n *= 3,624)	Removing those with high symptoms (EPDS > 12) antenatally^a^ (*n *= 3,073)	Adjusted for maternal depression at age 7, child IQ at age 8, maternal age, maternal education, social class, birth weight, gestation, and school enjoyment (*n *= 3,624)		
Measures of executive functions					
Working memory					
Nonword recall	−.027 (−.071 to .017) *p* = .232	.04 (−.02 to .09) *p* = .181	.02 (−.02 to .7) *p* = .311	−.09 (−.16 to −.03) *p* = .006	−.01 (−.04 to .03) *p* = .729
Digit span	.004 (−.04, .044) *p* = .864	−.01 (−.17, .15) *p* = .892	−.016 (−.07, .03) *p* = .502	−.042 (−.11, .02) *p* = .189	.02 (−.02, .06) *p* = .263
Attention switching					
Dual‐attention task	.05 (.01, .09) *p* = .008	.05 (<.01, .10) *p* = .049	.01 (−.012, .03) *p* = .361	.11 (.05, .17) *p* < .001	.044 (.01, .089) *p* = .050
Attentional control					
Inhibition task	.12 (.04, .20) *p* = .007	.12 (.01, .23) *p* = .039	.09 (.04, .14) *p* < .001	.11 (.025, .20) *p* = .011	.11 (.03, .19) *p* = .006
Measures of related cognitive abilities					
Selective attention					
Sky search	.02 (−.02, .06) *p* = .338	<.001 (−.04, .06) *p* = .700	.02 (−.02, .07) *p* = .368	.03 (−.03, .10) *p* = .304	.03 (−.01, .07) *p* = .100
Processing speed					
Verbal	.04 (.02, .06) *p* < .001	.04 (.01, .07) *p* = .023	.04 (.01, .06) *p* = .014	.05 (.01, .09) *p* = .014	.05 (.02, .07) *p* = .001
Motor	.03 (−.01, .08) *p* = .172	.02 (−.03, .8) *p* = .413	.04 (−.01, .10) *p* = .095	.07 (.004, .13) *p* = .036	.04 (−.01, .08) *p* = .061

a A sensitivity analysis was conducted removing those with antenatal depression to account for possible effects of antenatal depression.

b A measure of maternal IQ was only administered for those mothers who attended the ALSPAC teen focus clinics with their children when the children were 16 years old, using the adult WASI on a sample of 3,320 biological mothers (*M* total IQ score = 99, *SD *= 14), of this sample 1,471 offspring had data on all other measures.

**Table 2:** Association between postnatal depression (PND) and binary and continuous variables for math and English language GCSEs grades, before and after adjustments for (a) confounding variables and (b) imputing for missing data

Subject	Unadjusted associations between PND score and exam grades (*n *= 3,270)		Adjusted for maternal depression at age 7, child IQ at age 8, maternal age, maternal education, social class, birth weight, gestation, and school enjoyment at 8 (*n *= 3,270)		Postimputation analyses with adjustments including maternal IQ (*n *= 5,801)	
	Odds of failing grades for each five‐point increase in PND score using logistic regression	Linear association with each five‐point increase in PND score and continuous grade score (increase in one refers to a grade lower)	Odds of failing to achieve pass grades	Linear association with exam scores	Odds of failing to achieve pass grades	Linear association with exam scores
English	OR 1.12 (1.01, 1.25) *p* = .035	β* *= .07 (.01, .1) *p* = .016	OR 1.08 (.96, 1.21) *p* = .191	β* *= .03 (−.25, .09) *p* = .279	OR 1.04 (.95, 1.14) *p* = .413	β = .02 (−.03, .07) *p* = .437
Math	OR 1.19 (1.08, 1.30) *p* = .001	β = .17 (.10, .24) *p* = .001	OR 1.15 (1.08, 1.31) *p* = .001	β = .09 (.08, .21) *p* = .007	OR 1.13 (1.04, 1.24) *p* = .007	β = .08 (.02, .13) *p* = .010

#### PND and exams

As seen in Tables S1 and S2, PND was associated with increased risk of offspring failing in math and language. The associations between PND and math remained following adjustments for socioeconomic and family factors and following imputation for missing data. However, associations with language attenuated.

### Mediation: indirect pathways through EF

**Figure 2:**
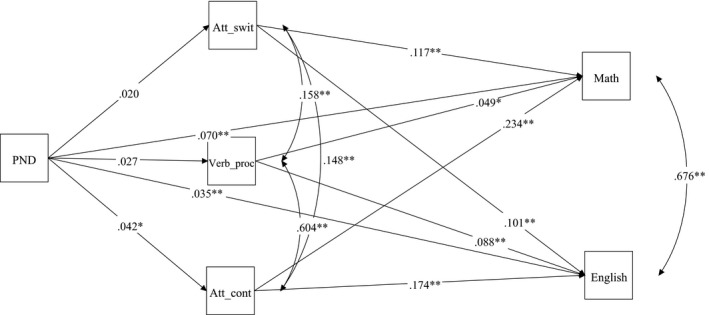
Each arrow represents a path with has been estimated using regression in Mplus, effect values on each path represent standardized path coefficients (these can be interpreted as correlations). Total effects represent the product of all pathway effect values from PND to Math (.083 (.012, .079) *p* < .001). Indirect effects represent the sum of the products of all indirect pathways {for example, the path coefficient for PND to EF factor *the path coefficient for EF to Math scores; [.013 (.003 to .028) *p* = .012] see Table S2}. Att_swit, attentional switching (dual task decrement score); Verb_proc, verbal processing (same worlds RT) and Att_cont, attentional control (opposite worlds RT). Curved arrows represent correlations between outcome variables. *p < 0.05; **p < 0.001

In the mutually adjusted path model (Figure 2), only evidence for an independent association between PND and attentional control emerged; the path coefficients from PND to switching and verbal processing attenuated (see Figure 2). The association between PND and exam grades was partially mediated: 27% (5%, 47%) of the association for English and 16% (4%, 27%) of the association for math were explained by indirect EF pathways (Figure 2 and Table S2, and Appendix S5).

### Prenatal effects

As shown in Table S3, there was evidence that both PND and antenatal anxiety were independently associated with math but not language. The association between PND and attentional control was also independent of antenatal anxiety. There was no evidence that antenatal anxiety was independently associated with attentional control or selective attention, but there was evidence that prenatal anxiety was associated with impaired working memory. There was no evidence for an independent effect of postnatal anxiety or prenatal anxiety in fathers on any of the outcomes.

**Figure 3:**
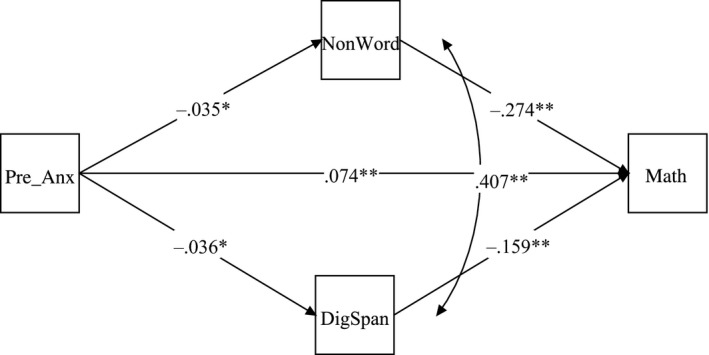
Mediation model from prenatal anxiety to math, through working memory. Each arrow represents a path with has been estimated using regression in Mplus, effect values on each path represent standardized path coefficients (these can be interpreted as correlations). Total effects represent the product of all pathway effect values (.090, *p* < .001). Indirect effects represent the sum of the products of all indirect pathways (.015, *p* = .006). The proportion of mediation is therefore indirect/total, .015/.090 = 17%. *p < 0.05; **p < 0.001. Pre_Anx, Antenatal anxiety; DigSpan, digit span score; NonWord, nonword recall score

Maternal anxiety was measured in pregnancy using the anxiety items from the Crown‐Crisp index, a validated self‐rating inventory (Birtchnell, Evans, & Kennard, 1988; Sutherland & Cooper, 1992). Given that we also found evidence for an independent association with prenatal anxiety and math, we explored whether impaired working memory mediated the association between antenatal anxiety and math. The mediation path analysis described above was repeated, replacing PND with antenatal anxiety (Figure 3). In this model 17% (13%–30%, *p* = .006) of the total association between prenatal anxiety and math was explained by indirect paths through working memory.

### Paternal comparisons

Logistic regression analyses provided evidence for an association between paternal PND and adolescents underachieving in math (OR to fail in math for a five‐point increase in postnatal paternal EPDS score: 1.16 (1.04 to 1.30, *p* = .007)). However, this effect diminished once maternal PND was included in the model (adjusted OR 1.09 (0.98 to 1.22, *p* = .078). In contrast, maternal PND associations were unaffected by the inclusion of paternal depression (adjusted OR 1.15 (1.04 to 1.27, *p* = .005)). There was no evidence that paternal depression was associated with English grades or any of the EFs. There was also no evidence that paternal anxiety in pregnancy was independently associated with math (adjusted OR 0.89, 0.80 to 1.0, *p* = .064) or any EF task. Because there was no evidence for main effects, we did not explore mediation.

## Discussion

To our knowledge this is the first large cohort‐study to investigate associations between maternal perinatal mental health and EFs in childhood or adolescent academic achievement at the end of high‐school. 36% of adolescents of persistently postnatally depressed mothers failed to ‘pass’ math at the end of high‐school compared to 27% of adolescents of nondepressed mothers. By this estimation, up to 8,100 more children in the United Kingdom would pass each year if we could increase the pass rate in offspring of depressed mothers to that of offspring of nondepressed mothers (Appendices S1–S6). A similar pattern was observed for language; however, there was evidence that this association was confounded by maternal IQ and education. There was also evidence for an independent association with maternal antenatal anxiety; however, antenatal anxiety and PND were associated with disruption of different aspects of EF, suggesting different pathways of risk for these related but distinct maternal risk factors. There was no evidence that paternal depression/anxiety was independently associated with adolescent exam grades, suggesting that the effects are specific to maternal mental health.

### Mechanisms

Observational studies alone do not provide causal evidence, and thus the mechanisms underlying observed associations remain to be established. However, some informed speculation is possible:

As with any association between maternal risk factors and child outcomes, a proportion of the association is likely to be explained by genetic and general environmental adversity (poorer living circumstances, lower education). However, in this case similar associations would be expected for paternal depression, which were not observed. Therefore, some discussion of nongenetic maternal specific effects is warranted.

There is increasing evidence from animals that antenatal stress leads to neurodevelopmental impairments in offspring, particularly working memory problems (Entringer et al., 2009). Therefore, a proportion of any association between maternal mental health and child cognitive outcomes may be explained by antenatal exposure. Indeed, here we report independent associations with antenatal anxiety and math. The specificity for math implies that these mechanisms disrupt specific skills. Consistent with previous studies (Buss et al., 2011), we found evidence that prenatal anxiety was specifically associated with difficulties in children's working memory.

However, we also report an association between PND and math following adjustments for antenatal anxiety, implying an additional role of postnatal exposure. This could be explained through links between PND and lack of parental stimulation, and reduced parental involvement in learning. On this argument, we would expect to see similar predictive effects for language and math. The independent effects for math specifically, points to disruption of more subtle cognitive abilities in the child. Indeed, PND was associated with impairments to a specific component of child EF, attentional control. As there was no evidence that antenatal anxiety was associated with impaired attentional control, antenatal effects on the attentional‐control pathway were unlikely.

Further work is needed to clarify how PND may be associated with reduced attentional control in children. However, two known consequences of PND could compromise the early development of attentional control:

1. A mother's contingent responsiveness to infant cues allows the young child to experience predictability and control of the environment and in turn learn how to effectively attend to environmental information (Baram et al., 2012). PND increases the risk that such parenting skills are impaired (Stein et al., 2014).
2. The child's attentional control also relies on the child developing capacities to self‐regulate ‘internal’ distractions, particularly emotional ones (Miyake & Friedman, 2012). In infancy, mother and infant emotion regulation are interdependent and form a basis from which the child learns self‐regulation (Choe, Olson, & Sameroff, 2013). However, depression compromises a mother's ability to regulate her own and her child's emotions (Joormann & Gotlib, 2010).

Early parenting‐programs which have been shown to improve the contingency and emotional sensitivity of parental responses (Jung, Short, Letourneau, & Andrews, 2007; Paris, Bolton, & Weinberg, 2009; Stein et al., 2006) could therefore promote the abilities of depressed mothers to facilitate their infant's development of attentional control.

Finally, maternal mental health is known to be associated with offspring emotional and behavioral problems (Stein et al., 2014). The role of such associations within the observed link between maternal mental health and child cognition is likely to be complex, and it is beyond the scope of the current paper. However, it is an important topic for future studies. Such problems in the child could contribute to the observed disruption to EF and math in offspring of depressed mothers, but such symptoms could also be a consequence of poorer cognition and achievement.

### Strengths and limitations

Strengths of this study include the large sample, long‐term follow‐up, objective laboratory cognitive measures at age 8, and external records of exam performance at age 16. The study design also allowed adjustment of key confounding variables and assessment of general cognitive abilities which could affect task performance.

That said, the associations obtained were generally of small effect size. However, given the dose–response relation, the 16‐year time frame, the population sample, and measurement error, all resulting in underestimation of any associations, such effects are likely to be meaningful. Small differences at a population level can greatly impact population health (Rose, 1985).

A relatively large proportion of data was missing. However, repeated measures allowed for multiple imputation techniques. In addition, there is considerable information on the pattern of missing data, which suggests that those most disadvantaged and more likely to have depressed mothers have missing data. This circumstance suggests that the current associations are likely underestimated, as those participants most likely to show positive associations are missing.

Depression scores were based on self‐report rather than clinical assessment. For mothers with persistently high scores, which have high specificity for diagnoses of depression, effect sizes were larger. This finding suggests that greater difficulties in both attentional‐control and academic achievement may be more likely in children of mothers with clinical depression.

There was no specific measure of maternal EFs, although we were able to account for maternal education and IQ. Future studies should therefore explore the role of maternal EF. That said, as there is no evidence that PND and antenatal anxiety are associated with different aspects of EF, the differential mediation we observed would seem unlikely if our results were explained by maternal EF alone. If inheritance of poorer EFs from parent to child explains the findings, we would also expect to see associations between paternal mental health and child EF, which was not the case in this study.

EF explained significant but small proportions of the associations between PND/antenatal anxiety and math. Inheritance of genetic vulnerabilities (Miyake & Friedman, 2012), shared adversity, as well as measurement error (see below) are all likely to contribute to the remaining pathways. Although the findings do not explain the full story, they identify specific and potentially modifiable pathways for intervention and highlight that the risk pathways from antenatal anxiety and PND could be different. At a population level, this distinction could be meaningful, particularly if EF can be modified at minimal cost.

In the current large population study, only one measure of each EF was available. As these measures were validated and reliable, this limitation does not invalidate our findings, but it is likely to have meant the effect sizes reported were underestimated. For example, there are several related but slightly different ways to measure EF. Previous evidence suggests that different tasks measuring the same component of EF are all highly correlated (see Miyake et al., 2012) and, thus, represent valid measures of the same underlying concept. However, each will involve different task demands. Some task demands will inevitably be unrelated to the specific EF component (e.g., speed to say words out loud or put pen to paper). If such demands differ in children with and without depressed mothers, this effect could account for observed associations. However, as we were able to adjust for IQ and processing speed, general performance factors which have also been linked to maternal depression were taken into account. Any remaining task differences are unlikely to be related to maternal depression and therefore likely represent random measurement error. Random measurement will always lead to underestimation of effect sizes. The ideal approach to reduce measurement error is to generate ‘latent’ factors for each EF from scores across more than one task. This approach separates concept‐relevant variance from task‐specific demands and as such reduces the impact of measurement error (Kline 2011, Pearson et al., 2015). Therefore, future studies should aim to replicate the findings in samples where it is possible to use multiple measures of EF.

### Implications

Maternal PND and antenatal anxiety are risk factors for adolescents under‐achieving in math exams at the end of high‐school. There is evidence for several intervention strategies (both psychological and pharmacological) which can effectively improve perinatal maternal mental health (Howard et al., 2014). Preventing, identifying, and treating maternal mental health in the perinatal period could, therefore, potentially increase adolescents’ chances of achieving in math. Early disruption to different aspects of EF explained a significant proportion of the risk to underachieving in math. If these pathways are causal, improving EF in children of depressed and anxious mothers could reduce their long‐term academic and professional/employment risk. Further work is needed to understand whether modifications to EF persist and transfer to academic functioning. However, there is promising evidence that EF can be modified in children through a variety of low‐cost approaches, including ‘Tools‐of‐the‐Mind’, family‐based training (Olds et al., 2014), exercise, martial‐arts, and mindfulness (Diamond & Lee, 2011).

**Key points**

- Achievement in math and English at the end of school is essential to health and success.
- Maternal mental health is associated with multiple negative child outcomes, but only a few studies have investigated how it impacts offspring leaving school grades.
- We report that prenatal anxiety and postnatal depression were independent predictors of poor achievement in math at age 16.
- Preventing, identifying, and treating maternal mental health issues in the perinatal period could, therefore, potentially increase adolescents’ chances of achieving in math.
- Early disruption to different aspects of EF explained a significant proportion of the risk to under‐achieving in math.
- If these pathways are causal, improving EF in children of depressed and anxious mothers could improve long‐term academic and professional/employment outcomes.

## Supporting information


**Appendix S1.** Exposure of maternal depression.
**Appendix S2.** Executive function measures.
**Appendix S3.** Mediation.
**Appendix S4.** Missing data.
**Appendix S5.** Mediation results.
**Appendix S6.** Discussion.
**Table S1.** Means of cognitive scores for EF and numbers of offspring failing in exams according to different PND groups.
**Table S2.** Total, direct and indirect, effects from postnatal depression (PND) to math and English language exam grades (based on model in Figure 2).
**Table S3.** Linear regression associations between continuous maternal symptom scores and EF, Math and English grades (*n *= 3,270).
