# Supplementary material for: Maternal perinatal mental health and offspring academic achievement at age 16: the mediating role of childhood executive function
Source: J Child Psychol Psychiatry. 2015 Nov 29;57(4):491–501. doi: 10.1111/jcpp.12483 (PMC4789117; doi:10.1111/jcpp.12483)
Supplement: Supplementary file 1 — Appendix S1. Exposure of maternal depression. Appendix S2. Executive function measures. Appendix S3. Mediation. Appendix S4. Missing data. Appendix S5. Mediation results. Appendix S6. Discussion. Table S1. Means of cognitive scores for EF and numbers of offspring failing in exams according to different PND groups. Table S2. Total, direct and indirect, effects from postnatal depression (PND) to math and English language exam grades (based on model in Figure 2). Table S3. Linear regression associations between continuous maternal symptom scores and EF, Math and English grades (n = 3,270). [file JCPP-57-491-s001.doc]

Supporting information for *Maternal perinatal mental health and offspring academic achievement at Age 16: the mediating role of childhood executive function* by Pearson et al.

**Appendix S1: Exposure of maternal depression**

EPDS scores > 12 have a high sensitivity and specificity in predicting clinically diagnosed depressive disorder, and scoring >12 on the EPDS on at least two occasions is considered to indicate depression likely to require treatment (Cox, 2003 ). We derived a 3-level categorical variable: 1) below threshold postnatally, 2) above threshold on the first EPDS only, and 3) above threshold on the first postnatal EPDS and above threshold on at least one occasion in the following waves (8 months, 1·5 years, 2·5 years), termed persisting/recurrent PND.

**Appendix S2: Executive function measures**

*Sky search task*

The child was presented with pictures of spaceships, informed that the spaceships always travel in pairs, and sometimes the pairs are identical and sometimes non-identical. The child’s task was to identify and circle the identical pairs as quickly as possible. This task was first demonstrated by the tester, the child had one practice sheet before completing the test sheet. In the test sheet, 20 (50%) of the spaceship pairs were identical. Reaction times in seconds to circle all of the spaceship pairs and number of correct pairs circled (out of 20) were recorded. As recommend in the TEA-Ch manual, an initial measure of ability on this task was taken as the reaction time to complete the test sheet (longer reaction times represent slower and thus worse ability) divided by the number of pairs correctly identified.

A measure of motor processing was also taken to account for the fact that some children may be quicker at circling spaceships due to motor control. Motor control was measured by repeating the task described with a new sheet containing only identical pairs. The time taken to complete this task served as a measure of motor processing. As recommend in the manual, motor processing reaction time was then subtracted from the ability score to provide our final measure of selective attention. *The higher the score, the more impaired the child’s selective attention (taking into account motor ability).*

*Switching*

As well as circling the identical spaceship pairs, the child counted how many noises were in each sequence and told the tester verbally at the end of sequence. An ability score for this version was calcualted from reaction time and accuracy to identify spaceships (as above), but this time the score was weighted by the child’s accuarcy in counting the noises. This score was then subtracted from the original selective attention score to provide an indication of the decrement in ability to do the task once the counting noise task was added. Again, a higher score indicates more impaired dual attention.

*Attentional control: opposite world task*

In this specific task, the child was shown a trail made up of the numbers 1 and 2 (with 24 numbers in total). In the ‘same world’ condition, he or she had to read the numbers out as quickly as possible (while the tester kept his or her finger next to each in the trail until the child had read it correctly). In the ‘opposite world’ condition, the child had to inhibit a pre-potent (very familiar) response and call out ‘two’ when he or she reached a 1 and ‘one’ when he or she reached a 2. The child was given a demonstration of each condition and had a practice attempt at each before being reminded of the rules. There were four test trials: a same world trial, followed by two opposite world trials, and finishing with another same world trial. During the task, the tester pointed to each digit in turn, only moving on to the next when a correct response was given, thus turning errors into a time penalty. The mean time taken to complete the same world trials was taken as a measure of verbal processing. The mean time on the opposite world trials was taken as the measure of attentional control: higher reaction times indicate more impaired ability.

**Appendix S3: Mediation**

Standardized path coefficients and standard errors were estimated using bootstrapping (5,000 models) in Mplus, using a maximum likelihood robust estimator. Bootstrapping is a non-parametric test; therefore, it does not rely on the assumptions of normality that are often not met when calculating indirect effects.

**Appendix S4: Missing data**

We employed a fully conditional specification as implemented using chained equations (the mi impute chained in STATA 12) using all variables described in the analyses and additional socio-demographic indicators of missingness as well as key predictors of missing exposure, confounding (including maternal IQ), and outcome data across 100 imputed datasets. Earlier measures of child cognitive abilities (IQ at several ages) and earlier academic achievement (national assessments: SATS at ages 7/8, 10/11, and 13/14) were used to predict missing EF data and GCSEs; allowing imputation to a sample with at least one of these scores (*n*=5,801). Monte Carlo errors were less than 10% of the standard error, and FMI values were no larger than 0.6..

**Appendix S5: Mediation results**

In a sensitivity analysis, we extended this model to the larger sample (n=5,801) using standard imputation techniques in Mplus and including the same auxiliary variables described above, also accounting for confounding.Following imputation and adjustment for confounding variables, all results were comparable: path coefficients of PND to attentional control, 0•046 (95% CI 0•018 to 0•073), and attentional control to English, 0•202 (95% CI 0•170 to 0•235) and to math, 0•130 (95% CI 0•097 to 0•167), with strong evidence for these indirect pathways (all ps<0•001).

**Appendix S6: Discussion**

*Calculation of impact of PND on passing Math in UK*

Based on approximately 600, 000 births in the UK per year, up to 15% of these will be affected by postnatal depression (90,000) and based on our current data, 36% of these children are predicted to fail at math (32,400). However, if the estimated failure rate was the same as for children of non-depressed mothers, 27% of these children would fail (24,300). Therefore if the risk associated with postnatal depression was removed 8,100 (32,400-24,300) less children would fail in the UK.

**Table S1 Means of cognitive scores for EF and numbers of offspring failing in exams according to different PND groups
(*on next page*)**

| **CONCEPT** | **COMPONENT** | **TEST** | **MEANS AND FREQUENCIES (SD)** | | | **STANDARDISED Β (95% CI)** | |
| --- | --- | --- | --- | --- | --- | --- | --- |
|  |  |  | **NO POSTNATAL DEPRESSION**  **(n=3459, 93%)** | **EARLY POSTNATAL EPISODE ONLY**  **(n=116, 3 %)** | **EARLY POSTNATAL EPISODE, RE-OCCURS**  **(N=164, 4%)** | **POSTNATAL EPISODE ONLY (COMPARED TO NO EPISODES)** | **POSTNATAL EPISODE, WHICH RE-OCCURS (COMPARED TO NO EPISODES)** |
| **EFs, AGE 8** | **Working Memory** | Non-word repetition  (accuracy score) | 7·2 (2) | 7·3(3) | 7·1 (2) | 0·001 (-0·15 to 0·17) *p=0*·*926* | -0·067 (-0·21 to 0·074) *p=0*·*355* |
|  |  |  |  |  |  |  |  |
|  |  | Digit span (accuracy score) | 10·3 (3) | 10·1 (3) | 9·9 (3) | -0·073 (0·24, 0·09) *p=0*·*384* | -0·14(-0·28 , 0·006) *p=0*·*061* |
|  | **Attention Switching** | Dual attention (sky search reaction time / accuracy to identify noises) | 5·6 (9) | 6·4 (10) | 6·4 (9) | 0·01 (-0·13 to 0·16) *p=0*·*858* | 0·19 (0·06 to 0·31) *p=0*·*004* |
|  |  |  |  |  |  |  |  |
|  | **Attentional-Control** | Inhibition task (‘opposite world’ reaction times in seconds) | 17·3 (3) | 17·9 (4) | 18·1 (4) | 0·18 (0·012, 0·34)  *p=0*·*035* | 0·23 (0·1, 0·4) *p=0*·*002* |
|  |  |  |  |  |  |  |  |
| **Related, COGNITIVE ABILITIES, AGE 8** | **Processing Speed** |  |  |  |  |  |  |
|  |  | Verbal (RT) (Reaction time to complete ‘same world’ condition, in seconds) | 13·0 (3) | 13·4 (3) | 13·5 (3) | 0·009 (-0·001, 0·18) *p=0*·*071* | 0·11 (0·03, 0·20) *p=0*·*006* |
|  |  | Motor (RT) (Reaction time on test sheet for sky search / number of correctly identified spaceships) | 1·4 (0·4) | 1·4 (0·4) | 1·4 (0·5) | 0·07 (-0·12, 0·24) *p=0*·*491* | 0·10 (-0·60, 0·26) *p=0*·*224* |
|  | **Selective Attention** | Selective attention (sky search reaction time in seconds, higher score is more impaired) | 5·1 (2) | 5·2 (2) | 5·2 (2) | 0·092 (-0·07, 0·25) *p=0*·*251* | 0·19 (-0·03, 0·24) *p=0*·*138* |
| **ACADEMIC ACHIEIVEMENT, AGE 16** | **GCSE Exams** |  |  |  |  | **OR (95% CI)** | **OR (95% CI)** |
|  |  | English Language Exam Grade  (% not reaching A*–C) | 23% | 26% | 24% | 1·19 (0·86 , 1·65) *p=0*·*304* | 1·10 (0·82, 1·42) *p=0*·*601* |
|  |  | Maths Exam Grade (% not reaching A*–C) | 27% | 29% | 36% | 1·11 (0·8, 1·53) *p=0*·*516* | 1·52 (1·19 ,1·94) *p=0*·*001* |

**Table S2: Total, direct and indirect, effects from postnatal depression (PND) to math and English language exam grades (based on the model in Figure 1).**

|  | **Total effects** (*combination of all pathways on figure 2*)  **N=3270** | **Indirect effects** (*product of specific individual pathways on figure 1*) | | | **Total indirect effects** (*combination of all indirect pathways on figure 2*) | **Proportion of total association explained by indirect EF pathways** *(indirect path coefficients /total effects*) |
| --- | --- | --- | --- | --- | --- | --- |
|  |  | **Attentional-Control** (*PND to Attentional Control * Attentional Control to Exam score)* | **Switching**  *(PND to Switching * Switching to Exam score*) | **Verbal Processing**  (*PND to Verbal Processing * Verbal Processing to Exam score)* |  |  |
| **Maths**  *Standardised Estimate (95% CI)* | 0·083 (0·012, 0·079) *p<0*·*001* | 0·010 (0·007, 0·018) *p<0*·*001* | 0·002 (-0·002, 0·003) *p=0*·*268* | 0·001 (-0·001, 0·003)  *p=0*·*224* | 0·013 (0·003 to 0·028) *p=0*·*012* | **16% (4%, 27%)** |
| **English**  *Standardised Estimate (95% CI)* | 0·046 (0·012, 0·079) *p=0*·*005* | 0·007 (0·001, 0·012) *p=0*·*025* | 0·002 (-0·002, 0·006) *p=0*·*268* | 0·002 (-0·002, 0·006) *p=0*·*224* | 0·012 (0·002 ,0·021) *p=0*·*014* | **27% (5% , 47%)** |

Note, effect values represent standardised path coefficients or products of the stated path coefficients.

| **Cognitive Outcomes** | **Associations with Postnatal Depression score**  **(adjusted for antenatal anxiety)**  **Standardised regression coefficients and 95% CI** | **Associations with Antenatal Anxiety score**  **(adjusted for postnatal depression)** |
| --- | --- | --- |
| Attentional Control | 0.06 (1.02 to 0.09) p=0.007 | 0.007 (-.02 to 0.04) p=0.615 |
| Attentional switching | 0.02 (-0.001 to 0.04) p=0.183 | -0.004 (-0.022 to 0.01) p=0.601 |
| Selective Attention | 0.04 (-0.002 to 0.083) p=0.067 | -0.01 (-0.04 to -0.02 ) P=0.527 |
| Working Memory  Digit Span  Non-word memory | 0.001 (-0.04 to 0.04) p=0.965  0.030 (-0.001 to 0.07) p=0.116 | -0.027 (-0.06 to 0.01) p=0.066  -0.035 (-0.06 to -0.006) p=0.017 |
| Failing Math | 1.09 (1.01 to 1.19) p=0.044 | 1.08 (1.01 to 1.14) p=0.014 |
| Failing English | 1.04 (0.94 to 1.14) p=0.434 | 1.06 (0.99 to 1.13) p=0.093 |

**Table S3** **Linear regression associations between continuous maternal symptom scores and EF, Math and English grades N=3270**
